# Long-Term Associations of a Mobile-Based Chronic Disease Management Program With Employee Health: Four-Year Retrospective Cohort Study

**DOI:** 10.2196/82822

**Published:** 2026-07-23

**Authors:** Yujin Park, Yoon Ji Kim, Boram Choi, Chang-bo Noh, Su Hwan Kim, Jiseon Bang, Ji-eun Lee, Jae-Heon Kang, Ye Seul Bae

**Affiliations:** 1 Healthcare Data Center, Kangbuk Samsung Hospital, Sungkyunkwan University School of Medicine, Seoul, Republic of Korea; 2 Big Data Research Center, Division of Healthcare Planning, Kangbuk Samsung Hospital, Sungkyunkwan University School of Medicine, Seoul, Republic of Korea; 3 Division of Healthcare Planning, Kangbuk Samsung Hospital, Sungkyunkwan University School of Medicine, Seoul, Republic of Korea; 4 Department of Information Statistics, Gyeongsang National University, Jinju-si, Gyeongsangnam-do, Republic of Korea; 5 Safety & Health Team, Global Manufacturing & Infra Technology, Samsung Electronics Co.Ltd., Hwaseong-si, Gyeonggi-do, Republic of Korea; 6 Department of Family Medicine, Kangbuk Samsung Hospital, Sungkyunkwan University School of Medicine, 29, Saemunan-ro, Jongno-gu, Seoul, Republic of Korea, 82 2-2001-1797, 82 2-734-2278; 7 Institute for Clinical Nutrition, Sungkyunkwan University School of Medicine, Seoul, Republic of Korea; 8 Institute for Future Healthcare, Sungkyunkwan University School of Medicine, Seoul, Republic of Korea

**Keywords:** mobile health, mHealth, chronic disease management, employee assistance program, EAP, digital health coaching, digital intervention

## Abstract

**Background:**

The burden of hypertension (HTN), type 2 diabetes mellitus (DM), and obesity is increasing among employees, driven by workplace-related factors such as sedentary behavior and unhealthy lifestyles, while follow-up management after health checkups remains inadequate. Mobile health (mHealth) interventions have emerged as promising tools to support self-management and sustain lifestyle changes; however, evidence regarding their long-term associations in real-world occupational settings remains limited.

**Objective:**

This study aimed to investigate whether a short-term mobile-based chronic disease management program was associated with sustained improvements in metabolic health outcomes among employees with metabolic risks over a 4-year period.

**Methods:**

The authors evaluated a 12-week mobile-based chronic disease management program delivered as part of an employee assistance program targeting workers with metabolic risk factors. Using propensity score–matched cohorts from the Kangbuk Samsung Health Study (HTN: N=90; DM: N=78; obesity: N=132), we analyzed longitudinal health examination data from 2019 (baseline) and 2023 (follow-up). Primary outcomes included blood pressure (BP), BMI, fasting glucose, glycated hemoglobin, triglycerides, and lipid profiles. Adjusted longitudinal changes were assessed using linear mixed effects models.

**Results:**

Among propensity score–matched cohorts, the intervention group showed more favorable changes compared to the controls. In the HTN cohort, systolic BP and diastolic BP showed no significant group × time interactions, although diastolic BP was lower in the intervention group at follow-up (*P*=.045). In the DM cohort, diastolic BP showed a significant group × time interaction (*P*=.02), with a decrease over time observed in the intervention group, while glycemic indicators did not differ significantly between groups. In the obesity cohort, triglycerides and high-density lipoprotein cholesterol showed significant group × time interactions (both *P*<.001), indicating more favorable longitudinal changes in the intervention group. Overall, the intervention groups demonstrated improved or stable cardiometabolic profiles compared to the control groups over the follow-up period.

**Conclusions:**

A short-term, personalized mHealth intervention was associated with favorable long-term changes over a 4-year follow-up period among employees with chronic disease risk. This real-world evidence supports integrating digital health programs into routine workplace prevention strategies to bridge the postcheckup care gap.

## Introduction

The increasing burden of noncommunicable diseases (NCDs), such as hypertension (HTN), type 2 diabetes mellitus (T2DM), and obesity, among employees represents a critical public health and economic challenge worldwide [1-3]. Employees may be at increased metabolic risk due to sedentary lifestyles, irregular eating habits, and chronic stress inherent in modern workplaces [4-7]. The increasing prevalence of NCDs among employees both threatens the health and productivity of individuals and results in significant costs to employers and health care systems [8].

In response to these challenges, mobile health (mHealth) interventions delivered through smartphone applications and wearable devices have emerged as promising tools for chronic disease management in occupational settings [9,10]. Growing evidence demonstrates that mHealth programs can improve clinical outcomes by supporting self-management, increasing medical adherence, and encouraging healthier behaviors [11,12]. A recent large-scale study of more than 1000 participants demonstrated significant improvements in metabolic indicators, including weight and glycemic control, following a 24-week mobile-based program [13]. In addition, randomized controlled trials have shown that mHealth interventions can improve short-term outcomes such as blood pressure (BP), glycemic control, and weight [14,15]. However, most existing studies have been limited to short-term follow-up periods of less than 1 year, leaving the long-term sustainability and real-world effectiveness of mHealth interventions insufficiently understood [16-18].

Many countries have implemented regular health checkups as a fundamental component of early detection and prevention of NCDs [1,19]. Korea, in particular, has a well-established and comprehensive health checkup system [20]. While these programs are effective in identifying at-risk individuals, follow-up management after screening often remains inadequate, limiting the benefits of early detection and opportunities for cost-effective secondary prevention [11,21]. Accordingly, there is growing interest in scalable and personalized intervention models that can more effectively support ongoing self-management and lifestyle modification [22].

To address this gap, this study evaluated the 4-year outcomes associated with a 12-week mobile-based chronic disease management program implemented by Kangbuk Samsung Hospital in 2021. The program was delivered as part of an employee assistance program (EAP) for a corporate partner, targeting employees with HTN, T2DM, or obesity-related metabolic risk identified through routine health checkups. Using propensity score–matched cohorts and longitudinal health checkup data collected before and after the intervention, this study aimed to assess whether customized digital health management can be associated with long-term improvements in key clinical indicators among employees. This study provides real-world evidence on the long-term durability and clinical utility of mHealth interventions in bridging the postcheckup care gap and supporting chronic disease prevention and management in workplace populations.

## Methods

### Data Source and Study Design

The Kangbuk Samsung Health Study is a large-scale retrospective cohort of adult male and female who have undergone comprehensive annual or biennial health checkups at the Kangbuk Samsung Hospital Total Healthcare Center in Seoul and Suwon, Korea [23]. Within this cohort, a 12-week mobile-based chronic disease management program was implemented in 2021, targeting employees identified as having HTN, T2DM, or obesity based on their 2019 health checkup, with participation being voluntary. This study focused on employees enrolled in a mobile chronic disease management program delivered as part of an EAP by Kangbuk Samsung Hospital and used their longitudinal health checkup data to assess long-term outcomes.

The initial population of 408,526 individuals who underwent at least 3 health checkups between 2002 and 2023 was further screened for eligibility. Eligible participants were those who completed at least 3 health checkups between 2002 and 2018 and who underwent follow-up health checkups in 2019 (baseline) and 2023 (follow-up). To ensure data consistency in measurement, health checkups from 2020, 2021, and 2022 were excluded from the analysis. This decision was based on changes in health checkup protocols and participation patterns during the COVID-19 pandemic that may have introduced systematic biases. During this period, social distancing policies and disruptions to routine health care services led to irregular participation in health checkups and an increased proportion of missing or incomplete data.

Based on these criteria, condition-specific cohorts were constructed for HTN, T2DM, and obesity, with participants classified into intervention and control groups according to enrollment in the mHealth program. Individuals with missing values in variables required for propensity score matching (PSM)—systolic blood pressure (SBP) and diastolic blood pressure (DBP), use of antihypertensive medication (for the HTN cohort), glycated hemoglobin (HbA_1c_), fasting blood glucose (FBG), use of antidiabetic medication (for the diabetes mellitus [DM] cohort), and BMI (for the obesity cohort)—were excluded. A 1:2 PSM procedure was then performed to construct comparable intervention and control groups.

### Ethical Considerations

This study was approved by the Institutional Review Board (IRB) of Kangbuk Samsung Hospital (IRB: KBSMC 2024-10-032). All intervention participants provided written informed consent for participation in the program, as well as for the use of their health checkup data for research purposes. For the control group, deidentified health checkup data were used, and the requirement for informed consent was waived by the IRB. All data were anonymized prior to analysis to ensure participant privacy and confidentiality, and access to the data was restricted to authorized researchers. Participants in the intervention program received small nonmonetary incentives (eg, thank-you gifts) upon completion of the program.

### Customized Mobile-Based Intervention

The intervention consisted of a structured 12-week mobile-based chronic disease management program delivered as part of an EAP to support workplace health promotion and chronic disease risk reduction. Employees identified through annual health checkups as having HTN, T2DM, or obesity-related metabolic risk were invited to enroll in 1 of 2 separate cycles between May 24 and December 26, 2021. At enrollment, participants underwent a preintervention physical and laboratory examination at the affiliated medical institution, provided informed consent, and received a dedicated health management mobile application. Participants were provided with a personalized care plan and Bluetooth-enabled self-monitoring devices (BP monitor, glucometer, or smart scale), depending on the specific disease management program in which they were enrolled.

During the intervention period, participants were encouraged to regularly record biometric and lifestyle data using a mobile application. Biometric data, including BP, blood glucose, and body weight, were automatically synchronized through connected devices, while lifestyle information such as diet, physical activity, and medication adherence was manually entered. These data were continuously transmitted to a web-based platform, allowing health care professionals to monitor participant status in real time. As the program was delivered through a mobile app, participants were able to actively self-monitor their health data, and real-time feedback from the health care coaching team further supported engagement and motivation for behavior change.

A multidisciplinary health coaching team consisting of nurses, clinical dietitians, and clinical exercise physiologists reviewed participant data weekly and provided tailored feedback based on the primary condition and real-time records. Feedback included health information messages, individualized commentary on submitted measurements, and guidance on behavior modification to improve metabolic risk factors. The intensity and frequency of this one-on-one feedback were adapted either automatically or manually according to individual risk levels and engagement, with more frequent monitoring and interaction provided for participants with higher clinical risk or lower adherence.

To enhance participant engagement and adherence, multiple supportive strategies were implemented. Participants received regular prompts and reminders through the mobile application to encourage consistent data entry and engagement. For individuals with low adherence, the health coaching team conducted follow-up contacts, such as phone calls, to promote continued participation.

**Figure 1.**
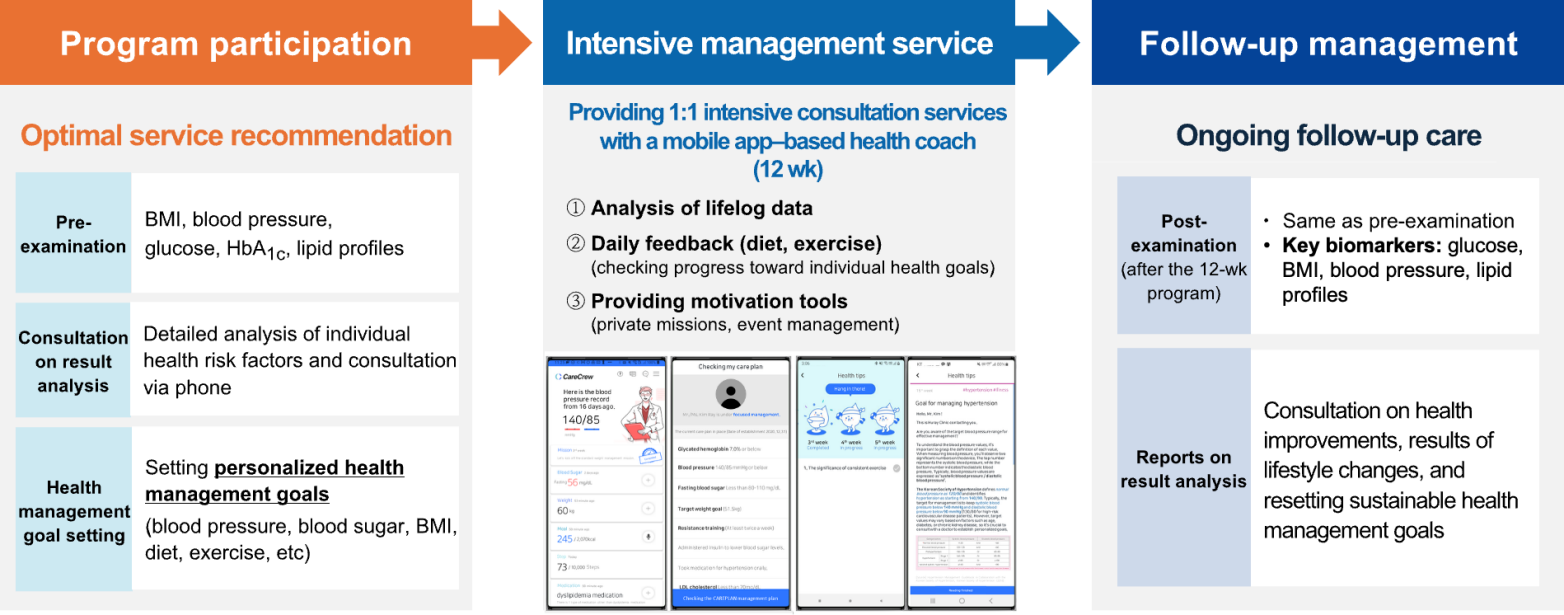
Framework of the customized mobile app–based intervention. HbA_1c_: glycated hemoglobin.

At program completion, participants underwent follow-up assessments equivalent to the preintervention examinations and received individualized reports summarizing their progress in clinical indicators. Aggregate results were shared with health coordinators and participants to inform ongoing health planning. On completing the entire process, participants received thank-you gifts as an additional incentive to encourage participation and retention (Figure 1).

### Outcome Measures

Although pre- and postprogram assessments were conducted as part of the 12-week intervention, this study focused on its long-term longitudinal associations observed after program participation. Outcomes were assessed by comparing health examination data from 2019 (baseline) and 2023 (follow-up), enabling the analysis of sustained changes beyond the immediate intervention period.

The primary outcomes were 9 clinical indicators commonly associated with chronic disease risk and management: SBP, DBP, BMI, FBG, HbA_1c_, triglycerides, total cholesterol, high-density lipoprotein (HDL) cholesterol, and low-density lipoprotein cholesterol. These biomarkers were selected based on both clinical judgment by specialists in family medicine and endocrinology and established clinical evidence, as they are essential indicators for HTN, DM, and obesity, as well as for overall cardiometabolic risk assessment and management [24-26].

In addition, short-term outcomes, including intervention-related survey responses and self-reported lifestyle behaviors assessed immediately before and after the 12-week intervention, were descriptively analyzed to complement the long-term findings. Satisfaction and perceived usefulness were assessed using 5 questionnaire items on a 5-point Likert scale, which were converted into a 100-point scale for analysis. Lifestyle behaviors were evaluated using self-reported questionnaires on smoking (average number of cigarettes per day), alcohol consumption (average number of drinks per day), and physical activity (total weekly exercise time).

### Statistical Analyses

To reduce confounding and improve comparability between the intervention and control groups, 1:2 PSM was performed using baseline data from 2019. Separate PSMs were estimated for each condition group based on their relevant covariates. The selection of matching variables was aligned with the real-world enrollment criteria of the intervention program, in which participants were identified based on key disease-specific clinical indicators. Age and sex were included as common covariates across all conditions. In addition, SBP, DBP, and use of antihypertensive medication were included for the HTN cohort; HbA_1c_, FBG, and use of antidiabetic medication were included for the DM cohort; and BMI was included for the obesity cohort.

A caliper width of 0.2 SD of the logit of the propensity score was applied for matching. Covariate balance before and after matching was assessed using absolute standardized mean differences (ASDs), with values less than 0.1 indicating adequate balance.

To compare longitudinal changes in outcomes associated with program participation, linear mixed effects models were applied separately within each HTN, DM, and obesity cohort. Fixed effects included group (intervention vs control), time (baseline vs follow-up), and their interaction (group × time), while a random intercept was included for each participant. The models were adjusted for baseline values and covariates with residual imbalance after PSM (ASDs≥0.1) within each cohort. Estimated marginal means were calculated to obtain adjusted group-specific outcomes at each time point, and the primary inference was based on the group × time interaction term, representing differences in longitudinal changes between groups. As a supplementary analysis, conventional statistical tests, such as paired and independent comparisons based on group comparisons, were also performed. Statistical significance was determined by a 2-sided *P*<.05, and all analyses were performed using R software version 4.3.1 (R Core Team).

## Results

### Study Population

**Figure 2.**
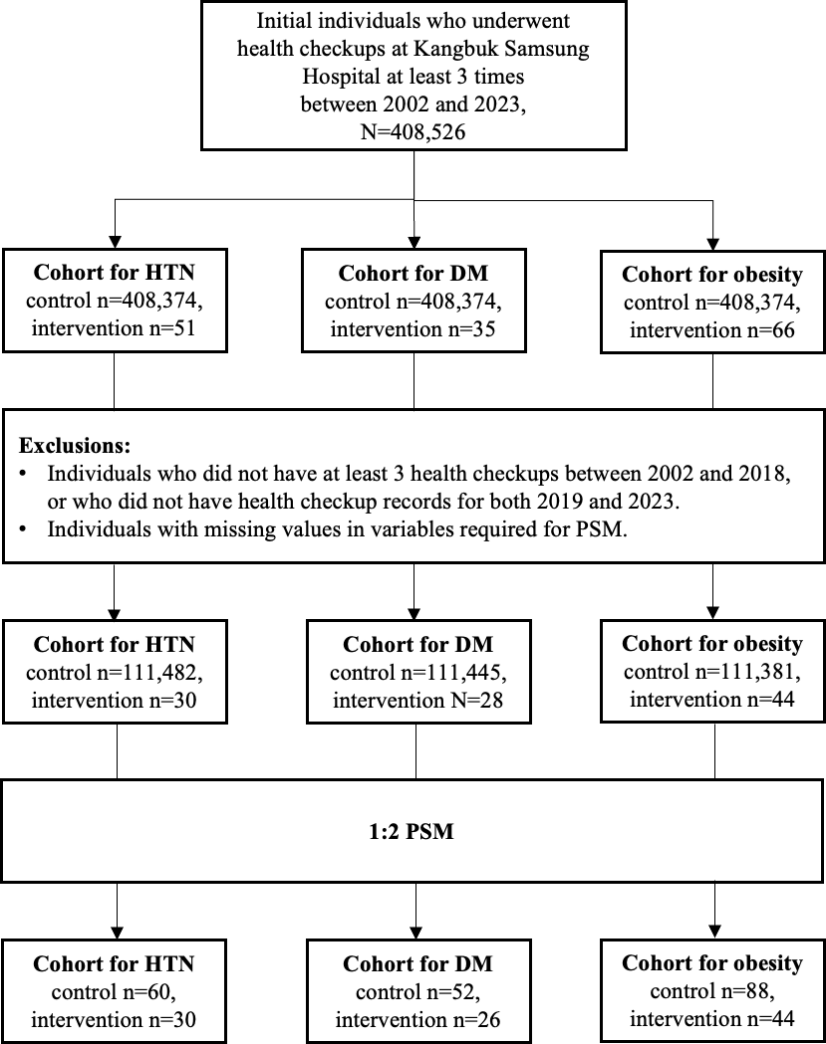
Flowchart of the study population. DM: diabetes mellitus; HTN: hypertension; PSM: propensity score matching.

The prematching cohorts included 30 intervention and 111,482 control individuals for HTN, 28 intervention and 111,445 control individuals for DM, and 44 intervention and 111,381 control individuals for obesity. After 1:2 PSM, the final analytical cohorts comprised 30 intervention and 60 control individuals for HTN, 26 intervention and 52 control individuals for DM, and 44 intervention and 88 control individuals for obesity (Figure 2).

### Covariate Balance Between Groups Before and After PSM

Table S1 in Multimedia Appendix 1 presents the covariate balance between the intervention and control groups before and after PSM across the HTN, DM, and obesity cohorts, focusing on the variables used for PSM. Prior to matching, there were substantial baseline imbalances in several key covariates between the groups, as evidenced by high ASDs, which indicate the degree of dissimilarity between groups. In this study, we considered an ASD of 0.1 or greater to indicate significant imbalance between groups.

In the HTN cohort, significant differences were observed in SBP and DBP, with ASDs of 0.755 and 1.013, respectively. These values reflect considerable baseline differences in BP control between the intervention and control groups. Similarly, the DM cohort showed ASDs with FBG and HbA_1c_ exceeding 1, highlighting a large difference in glycemic status prior to matching. The obesity cohort showed a slightly more modest imbalance, but BMI still had an ASD of 0.388, suggesting unequal body composition.

After applying 1:2 PSM, intervention participants were successfully matched to comparable controls from a large eligible pool, resulting in improved balance in key covariates. Notably, all ASDs were reduced below the threshold of 0.1 across all measured covariates. In many cases, the postmatching ASDs approached zero, reflecting near-perfect alignment of baseline characteristics between the groups.

### Baseline Characteristics of Primary Outcomes for Matched Cohorts

Table 1 summarizes the baseline distribution of key clinical outcomes for the matched intervention and control groups across the HTN, DM, and obesity cohorts. Notably, SBP and DBP were well-aligned in both the HTN and obesity cohorts, with ASDs close to 0. In addition, FBG and HbA_1c_ were consistently balanced across all 3 cohorts, reflecting comparable glycemic profiles at baseline.

**Table 1.** Baseline characteristics of primary outcomes for matched cohorts.

Characteristics	Hypertension (N=90)			Diabetes mellitus (N=78)			Obesity (N=132)		
	Intervention (n=30)	Control (n=60)	ASD^a^	Intervention (n=26)	Control (n=52)	ASD	Intervention (n=44)	Control (n=88)	ASD
Age (y), mean (SD)	46.10 (4.76)	46.10 (4.70)	0	46.85 (4.33)	46.85 (4.20)	0	45.93 (4.74)	45.93 (4.71)	0
Sex, n (%)			0			0			0
Female	1 (3.33)	2 (3.33)		1 (3.85)	2 (3.85)		3 (6.82)	6 (6.82)	
Male	29 (96.67)	58 (96.67)		25 (96.15)	40 (96.15)		41 (93.18)	82 (93.18)	
SBP^b^ (mm Hg), mean (SD)	119.53 (12.12)	119.57 (11.92)	0.003	117.50 (11.09)	114.27 (10.31)	0.306	111.68 (10.06)	112.22 (10.26)	0.052
DBP^c^ (mm Hg), mean (SD)	81.67 (9.09)	81.67 (9.01)	0	77.46 (7.84)	75.88 (7.76)	0.203	74.52 (8.03)	74.65 (8.71)	0.015
FBG^d^ (mg/dL), mean (SD)	100.77 (8.16)	100.83 (11.75)	0.006	110.81 (16.02)	110.77 (15.79)	0.004	100.68 (10.22)	101.82 (13.34)	0.092
HbA_1c_^e^ (%), mean (SD)	5.54 (0.30)	5.52 (0.40)	0.067	5.98 (0.49)	5.99 (0.49)	0.002	5.57 (0.40)	5.58 (0.46)	0.026
BMI (kg/m^2^), mean (SD)	26.04 (3.80)	25.04 (3.22)	0.295	26.99 (2.67)	25.17 (2.62)	0.690	25.17 (2.06)	25.17 (2.05)	0.001
Triglycerides (mg/dL), mean (SD)	158.57 (76.74)	152.97 (76.35)	0.073	158.23 (84.33)	180.17 (86.27)	0.256	172.80 (91.11)	142.39 (89.49)	0.338
Total cholesterol (mg/dL), mean (SD)	189.33 (37.58)	194.18 (30.29)	0.148	194.23 (43.08)	201.42 (35.55)	0.188	207.59 (34.40)	191.02 (33.30)	0.492
HDL^f^ cholesterol (mg/dL), mean (SD)	55.50 (15.63)	52.95 (14.10)	0.174	46.96 (11.24)	51.62 (12.00)	0.396	53.36 (14.29)	54.59 (14.06)	0.087
LDL^g^ cholesterol (mg/dL), mean (SD)	125.10 (32.54)	133.35 (28.52)	0.276	137.96 (42.62)	139.44 (36.56)	0.038	142.32 (30.35)	130.01 (32.37)	0.388

a ASD: absolute standardized mean difference.

b SBP: systolic blood pressure.

c DBP: diastolic blood pressure.

d FBG: fasting blood glucose.

e HbA_1c_: glycated hemoglobin.

f HDL: high-density lipoprotein.

g LDL: low-density lipoprotein.

Despite improvements in key disease-specific indicators, several variables showed persistent imbalances across multiple cohorts. Total cholesterol exhibited significant differences between groups in all 3 cohorts (ASD=0.148 in HTN, 0.188 in DM, and 0.492 in obesity). BMI also demonstrated notable imbalances in both HTN (ASD=0.295) and DM (ASD=0.690) cohorts.

While PSM successfully achieved balance in key disease-specific indicators within each cohort, residual differences in certain baseline characteristics, particularly lipid-related and anthropometric variables, persisted across cohorts. To account for these residual imbalances, variables with an ASD≥0.1 were included as covariates in the subsequent linear mixed effects models.

### Adjusted Longitudinal Changes in Primary Clinical Outcomes Across Matched Cohorts

Table 2 presents longitudinal changes in key clinical indicators based on linear mixed effects models using estimated marginal means across the matched HTN, DM, and obesity cohorts. Baseline values were well balanced between the intervention and control groups across all outcomes.

**Table 2.** Estimated marginal means (EMMs) and longitudinal changes in primary outcomes in intervention and control groups across cohorts^a^.

Characteristics	Hypertension (N=90)			Diabetes mellitus (N=78)			Obesity (N=132)		
	Intervention (n=30)	Control (n=60)	*P* value	Intervention (n=26)	Control (n=52)	*P* value	Intervention (n=44)	Control (n=88)	*P* value
SBP^b^ (mm Hg), EMM (SE)									
2019	119.50 (1.67)	119.59 (1.17)	.97	115.30 (1.53)	115.37 (1.06)	.97	111.81 (1.10)	112.15 (0.77)	.80
2023	119.60 (1.67)	122.04 (1.17)	.24	113.42 (1.53)	117.68 (1.06)	.03**^c^**	111.97 (1.10)	113.98 (0.77)	.14
*P* for interaction			.41			.10			.38
DBP^d^ (mm Hg), EMM (SE)									
2019	81.75 (1.09)	81.64 (0.76)	.93	76.25 (1.10)	76.49 (0.77)	.86	74.58 (0.80)	74.62 (0.56)	.97
2023	76.61 (1.09)	79.31 (0.76)	.045**^c^**	71.71 (1.10)	76.38 (0.77)	<.001**^c^**	71.99 (0.80)	73.69 (0.56)	.09
*P* for interaction			.13			.02**^c^**			.23
FBG^e^ (mg/dL), EMM (SE)									
2019	100.86 (1.01)	100.78 (0.71)	.95	109.95 (2.45)	111.20 (1.70)	.68	100.98 (1.79)	101.67 (1.26)	.76
2023	97.93 (1.01)	97.35 (0.71)	.74	110.99 (2.45)	111.52 (1.70)	.86	98.14 (1.79)	99.57 (1.26)	.52
*P* for interaction			.77			.86			.81
HbA_1c_^f^ (%), EMM (SE)									
2019	5.53 (0.03)	5.52 (0.02)	.73	5.97 (0.08)	5.99 (0.06)	.82	5.57 (0.04)	5.58 (0.03)	.83
2023	5.59 (0.03)	5.64 (0.02)	.18	6.13 (0.08)	6.18 (0.06)	.72	5.66 (0.04)	5.70 (0.03)	.44
*P* for interaction			.22			.92			.70
BMI (kg/m^2^), EMM (SE)									
2019	25.42 (0.18)	25.35 (0.12)	.75	25.88 (0.20)	25.72 (0.14)	.52	25.17 (0.13)	25.17 (0.09)	.97
2023	25.17 (0.18)	25.21 (0.12)	.85	25.84 (0.20)	25.37 (0.14)	.06	25.34 (0.13)	25.36 (0.09)	.90
*P* for interaction			.71			.36			.91
Triglycerides (mg/dL), EMM (SE)									
2019	154.47 (10.69)	155.01 (7.52)	.97	166.82 (10.08)	175.88 (7.01)	.47	158.02 (8.15)	149.78 (5.73)	.41
2023	156.37 (10.69)	151.83 (7.52)	.73	140.12 (10.08)	141.48 (7.01)	.91	117.81 (8.15)	157.74 (5.73)	<.001**^c^**
*P* for interaction			.78			.65			<.001**^c^**
Total cholesterol (mg/dL), EMM (SE)									
2019	191.53 (5.09)	193.08 (3.58)	.80	197.16 (5.97)	199.96 (4.15)	.71	199.93 (4.09)	194.85 (2.88)	.32
2023	182.86 (5.09)	196.80 (3.58)	.03**^c^**	180.66 (5.97)	193.36 (4.15)	.09	195.81 (4.09)	195.42 (2.88)	.94
*P* for interaction			.16			.33			.51
HDL^g^ cholesterol (mg/dL), EMM (SE)									
2019	53.78 (1.15)	53.81 (0.81)	.99	49.71 (1.29)	50.24 (0.89)	.74	54.15 (0.85)	54.20 (0.60)	.96
2023	56.15 (1.15)	57.26 (0.81)	.44	54.59 (1.29)	54.61 (0.89)	.99	59.96 (0.85)	54.88 (0.60)	<.001**^c^**
*P* for interaction			.59			.81			<.001**^c^**
LDL^h^ cholesterol (mg/dL), EMM (SE)									
2019	129.46 (4.68)	131.17 (3.29)	.77	138.55 (5.81)	139.15 (4.03)	.93	136.44 (3.77)	132.95 (2.65)	.45
2023	114.33 (4.68)	126.18 (3.29)	.04**^c^**	117.13 (5.81)	126.59 (4.03)	.19	127.30 (3.77)	125.26 (2.65)	.66
*P* for interaction			.21			.36			.82

a EMMs were calculated from linear mixed effects models adjusted for covariates with residual imbalance (absolute standardized mean difference ≥0.1) after propensity score matching.

b SBP: systolic blood pressure.

c Statistically significant results (*P*<.05).

d DBP: diastolic blood pressure.

e FBG: fasting blood glucose.

f HbA_1c_: glycated hemoglobin.

g HDL: high-density lipoprotein.

h LDL: low-density lipoprotein.

In the HTN cohort, SBP levels were comparable between groups at both baseline (2019) and follow-up (2023), and the group × time interaction was not statistically significant (*P*=.41), indicating no differential change over time. In contrast, DBP was significantly lower in the intervention group compared to the control group at follow-up (76.61 vs 79.31 mm Hg; *P*=.045); however, the interaction term was not significant (*P*=.13), suggesting that although a between-group difference was observed in 2023, the overall longitudinal change pattern did not differ significantly between groups.

In the DM cohort, SBP was significantly lower in the intervention group compared to the control group at follow-up (113.42 vs 117.68 mm Hg; *P*=.03), although the interaction term was not statistically significant (*P*=.10), indicating a trend without a significant difference in change patterns. Notably, DBP showed both a significant between-group difference at follow-up (71.71 vs 76.38 mm Hg; *P*<.001) and a significant group × time interaction (*P*=.02), with a decrease over time observed in the intervention group. Other glycemic indicators, including FBG and HbA_1c_, did not show significant differences in either between-group comparisons or interaction terms.

In the obesity cohort, triglycerides showed a significant between-group difference at follow-up (117.81 vs 157.74 mg/dL; *P*<.001), along with a significant interaction (*P*<.001), suggesting a favorable change pattern associated with the intervention. Similarly, HDL cholesterol was significantly higher in the intervention group at follow-up (59.96 vs 54.88 mg/dL; *P*<.001), with a significant interaction term (*P*<.001), suggesting a favorable change pattern associated with the intervention.

Analyses based on observed (unadjusted) values, including within- and between-group comparisons, yielded generally consistent patterns and are presented in Table S2 in Multimedia Appendix 1.

### Short-Term Behavior Changes and Intervention-Related Survey Results

Survey responses collected immediately after the intervention indicated high levels of satisfaction and perceived usefulness of the program, with mean scores of 83.8 and 83.7, respectively.

Consistent with these findings, self-reported lifestyle behaviors assessed immediately before and after the intervention showed changes across multiple domains. In the HTN, DM, and obesity cohorts, some participants reported reductions in cigarette consumption after the intervention. Alcohol intake demonstrated mixed patterns, with a greater proportion of participants reporting decreases rather than increases. Physical activity distributions shifted toward higher activity categories, with an increased number of participants achieving ≥180 minutes per week compared to preintervention levels (Table S3 in Multimedia Appendix 1).

## Discussion

### Overview of Key Findings and Clinical Relevance

This study provides preliminary real-world evidence suggesting long-term associations between participation in a relatively short-term digital health intervention delivered as part of an EAP and several cardiometabolic indicators across multiple chronic disease risk groups over 4 years of follow-up. The intervention group was associated with favorable changes in several cardiometabolic indicators, particularly in DBP, triglycerides, and HDL cholesterol, based on adjusted longitudinal analyses. Although some statistically significant changes were modest in magnitude, even small changes in these clinically relevant cardiometabolic markers may be meaningful at the population level [27-29].

In the HTN cohort, while cross-sectional differences in DBP were observed at follow-up, there was insufficient evidence of significantly different DBP trajectories over time between the groups. In contrast, in the DM cohort, DBP showed both a significant between-group difference at follow-up and a significant group × time interaction, indicating different longitudinal DBP trajectories between the groups. These findings are broadly consistent with previous studies reporting associations between mHealth-based interventions and BP control over time [30,31].

In the obesity cohort, triglycerides and HDL cholesterol showed the most pronounced favorable changes in the intervention group, with both significant between-group differences at follow-up and longitudinal change patterns. These observed lipid changes are clinically relevant, as triglycerides and HDL cholesterol are established markers associated with cardiovascular risk [32,33].

### Potential Mechanisms Underlying Sustained Associations

Although this study did not directly evaluate behavioral or engagement mechanisms, several hypothesized pathways may help explain the observed associations. Participants reported high levels of satisfaction and perceived usefulness during the intervention period, along with favorable short-term changes in lifestyle behaviors. Previous studies have suggested that mHealth interventions combining real-time self-monitoring, personalized feedback, and behavioral coaching may support long-term adherence and lifestyle modification [34-38]. However, because detailed app usage metrics and longitudinal behavioral adherence data were not systematically collected in this study, these proposed mechanisms should be considered exploratory and were not directly tested in this analysis.

### Implications for Health Care Systems and Workplace-Based Interventions

From a health care system perspective, the findings suggest that workplace-based mHealth programs may serve as a scalable complement to existing chronic disease management infrastructure. In countries with established national health checkup systems, integrating digital support into employee health programs may help strengthen long-term follow-up and secondary prevention after health screenings [13,18,21]. In addition, mHealth platforms may help reduce logistical and psychological barriers to ongoing disease management by providing continuous support integrated into daily routines, rather than relying solely on episodic clinic-based care [39]. These findings are also consistent with growing international interest in digital therapeutics and reimbursement models for mHealth-based prevention strategies targeting HTN, DM, and obesity [1,40,41]. In occupational settings, digital interventions embedded within EAPs may improve accessibility and continuity of care for working populations with limited opportunities for regular in-person follow-ups [2,10,12]. Collectively, these findings support the potential role of workplace-integrated digital health programs in chronic disease prevention and management.

### Limitations

Several limitations should be acknowledged. First, the retrospective nature of the analysis and the limited program cycle resulted in a relatively small sample size with complete follow-up data in 2023. This may reduce the statistical power of the study and increase the likelihood of type II errors, particularly for outcomes with relatively small effect sizes or nonsignificant longitudinal trends. Therefore, the findings should be interpreted cautiously, and the observed associations require further validation in larger prospective studies. Second, selection bias cannot be completely ruled out, as participants may have been more motivated or health-literate than the general population. Although PSM and linear mixed effects models were used to balance clinical variables, unmeasured confounders such as health behaviors, income, or education level may still have influenced the results. In addition, several baseline variables, including BMI and lipid-related markers, remained partially imbalanced after matching. Although these variables were additionally adjusted for in the longitudinal models, residual imbalance may still limit the strength of causal interpretation. Third, the absence of intermediate assessments between baseline (2019) and follow-up (2023) limits our ability to assess the trajectory of behavioral maintenance or identify when improvements plateaued or declined. As a result, the observed long-term outcomes should be interpreted as associations rather than direct causal effects of the intervention. Additionally, although short-term survey-based assessments provided some indication of behavior changes following the intervention, the absence of detailed and systematically collected app usage metrics (eg, log-ins, device adherence, coaching frequency) precluded detailed dose-response analyses of the underlying mechanisms. Finally, the generalizability of the findings may be limited, as the study population was derived from a single corporate system in Korea and consisted predominantly of male office workers.

### Future Directions

Based on the findings and limitations of this study, future research should investigate the active elements of digital interventions—such as feedback frequency, personalization of advice, accountability of self-tracking, or credibility of professional coaching—that most effectively drive long-term behavior change. Furthermore, prospective studies with larger, more diverse cohorts are needed to generalize these results and investigate the compounding effects of cumulative interventions. Linking digital intervention data with claims databases or electronic health records would also enable assessments of downstream health care utilization and cost-effectiveness. Additionally, incorporating adaptive AI-based personalization, nudge algorithms, or gamification could enhance engagement, particularly among younger or more tech-savvy populations. Ultimately, future mHealth programs should aim to create hybrid models that combine medical, behavioral, and digital precision.

## Supplementary material

10.2196/82822 Multimedia Appendix 1 Table S1: Covariate balance before and after propensity score matching for each condition; Table S2: Observed clinical outcomes at baseline and follow-up in the matched intervention and control groups; and Table S3: short-term changes in lifestyle behaviors between baseline and follow-up in the intervention group.

## Abbreviations

ASD: absolute standardized mean difference
BP: blood pressure
DBP: diastolic blood pressure
DM: diabetes mellitus
EAP: employee assistance program
FBG: fasting blood glucose
HbA_1c_: glycated hemoglobin
HDL: high-density lipoprotein
HTN: hypertension
IRB: institutional review board
mHealth: mobile health
NCD: noncommunicable disease
PSM: propensity score matching
SBP: systolic blood pressure
T2DM: type 2 diabetes mellitus
