# Supplementary material for: Long-Term Associations of a Mobile-Based Chronic Disease Management Program With Employee Health: Four-Year Retrospective Cohort Study
Source: J Med Internet Res. 2026 Jul 23;28:e82822. doi: 10.2196/82822 (PMC13394854; doi:10.2196/82822)
Supplement: Multimedia Appendix 1 [file jmir-v28-e82822-s001.docx]

**Supplementary Table S1. Covariate balance before and after propensity score matching for each condition**

|  | **Before matching** | | | |  | **After matching** | | | |
| --- | --- | --- | --- | --- | --- | --- | --- | --- | --- |
|  | **Overall** | **Control** | **Inter-vention** | **ASD** |  | **Overall** | **Control** | **Inter-vention** | **ASD** |
| **Hypertension** | | | | |  |  | | | |
| N | 111,512 | 111,482 | 30 |  |  | 90 | 60 | 30 |  |
| Age | 44.12 (5.93) | 44.12 (5.93) | 46.10 (4.76) | 0.335 |  | 46.10 (4.69) | 46.10 (4.70) | 46.10 (4.76) | 0 |
| Sex (%) |  |  |  | 1.011 |  |  |  |  | 0 |
| Female | 45,401 (40.71) | 45,400 (40.72) | 1 (3.33) |  |  | 3 (3.33) | 2 (3.33) | 1 (3.33) |  |
| Male | 66,111 (59.29) | 66,082 (59.28) | 29 (96.67) |  |  | 87 (96.67) | 58 (96.67) | 29 (96.67) |  |
| Systolic BP | 110.15 (12.43) | 110.14 (12.43) | 119.53 (12.12) | 0.755 |  | 119.56 (11.92) | 119.57 (11.92) | 119.53 (12.12) | 0.003 |
| Diastolic BP | 71.77 (9.77) | 71.77 (9.77) | 81.67 (9.09) | 1.013 |  | 81.67 (8.98) | 81.67 (9.09) | 81.67 (9.01) |  |
| Medication (%) |  |  |  | 0.388 |  |  |  |  | 0 |
| No | 103,704 (93.00) | 103,674 (93.00) | 30 (100.00) |  |  | 90 (100.00) | 60 (100.00) | 30 (100.00) |  |
| Yes | 7,808 (7.00) | 7,808 (7.00) | 0 (0.00) |  |  | 0 (0.00) | 0 (0.00) | 0 (0.00) |  |
| **Diabetes mellitus** | | | | |  |  | | | |
| N | 111,473 | 111,445 | 28 |  |  | 78 | 52 | 26 |  |
| Age | 44.12 (5.93) | 44.12 (5.93) | 47.04 (4.23) | 0.492 |  | 46.85 (4.22) | 46.85 (4.20) | 46.85 (4.33) | 0 |
| Sex (%) |  |  |  | 1.000 |  |  |  |  | 0 |
| Female | 45,366 (40.70) | 45,365 (40.71) | 1 (3.57) |  |  | 3 (3.85) | 2 (3.85) | 1 (3.85) |  |
| Male | 66,107 (59.30) | 66,080 (59.29) | 27 (96.43) |  |  | 75 (96.15) | 50 (96.15) | 25 (96.15) |  |
| FBG | 99.42 (15.60) | 99.41 (15.60) | 117.18 (28.54) | 1.139 |  | 110.78 (15.76) | 110.77 (15.79) | 110.81 (16.02) | 0.002 |
| HbA1c | 5.54 (0.54) | 5.54 (0.54) | 6.27 (1.25) | 1.333 |  | 5.99 (0.49) | 5.99 (0.49) | 5.98 (0.49) | 0.004 |
| Medication (%) |  |  |  | 0.055 |  |  |  |  | 0 |
| 0 | 108,561 (97.39) | 108,534 (97.39) | 27 (96.43) |  |  | 78 (100.00) | 52 (100.00) | 26 (100.00) |  |
| 1 | 2,912 (2.61) | 2,911 (2.61) | 1 (3.57) |  |  | 0 (0.00) | 0 (0.00) | 0 (0.00) |  |
| **OB** | | | | |  |  | | | |
| N | 111,425 | 111,381 | 44 |  |  | 132 | 88 | 44 |  |
| Age | 44.13 (5.93) | 44.13 (5.93) | 45.93 (4.74) | 0.305 |  | 45.93 (4.70) | 45.93 (4.71) | 45.93 (4.74) | 0 |
| Sex (%) |  |  |  | 0.867 |  |  |  |  | 0 |
| Female | 45,294 (40.65) | 45,291 (40.66) | 3 (6.82) |  |  | 9 (6.82) | 6 (6.82) | 3 (6.82) |  |
| Male | 66,131 (59.35) | 66,090 (59.34) | 41 (93.18) |  |  | 123 (93.18) | 82 (93.18) | 41 (93.18) |  |
| BMI | 23.87 (3.42) | 23.87 (3.42) | 25.17 (2.06) | 0.380 |  | 25.17 (2.04) | 25.17 (2.05) | 25.17 (2.06) | 0.001 |

* Data are presented as means (standard deviations) or numbers (percentages).
* Abbreviations: BP: blood pressure; FBG: fasting blood glucose; HbA1c: hemoglobin A1c; BMI: body mass index; ASD: absolute standardized mean difference.

**Supplementary Table S2. Observed clinical outcomes at baseline (2019) and follow-up (2023) in the matched intervention and control groups**

|  |  | **Hypertension (N=90)** | | | **Diabetes mellitus (N=78)** | | | **Obesity (N=132)** | | |
| --- | --- | --- | --- | --- | --- | --- | --- | --- | --- | --- |
|  |  | **Intervention (N=30)** | **Control (N=60)** | ***p* value** | **Intervention (N=26)** | **Control (N=52)** | ***p* value** | **Intervention (N=44)** | **Control (N=88)** | ***p* value** |
| **SBP, mean (mmHg)** | **2019** | 119.53$\pm$12.12 | 119.57$\pm$11.92 | 0.99^1^ | 117.50$\pm$11.09 | 114.27$\pm$10.31 | 0.21^1^ | 111.68$\pm$10.06 | 112.22$\pm$10.26 | 0.78^1^ |
|  | **2023** | 119.63$\pm$14.55 | 122.02$\pm$13.94 | 0.28^3^ | 115.62$\pm$12.22 | 116.58$\pm$10.17 | 0.73^1^ | 111.84$\pm$9.34 | 114.05$\pm$10.78 | 0.24^3^ |
|  | **Diff.** | 0.10 | 2.45 | <0.001^2^ | -1.88 | 2.31 | <0.001^1^ | 0.16 | 1.83 | <0.001^1^ |
|  | ***p* value** | 0.85^3^ | 0.120^1^ |  | 0.48^1^ | 0.27^1^ |  | 0.92^1^ | 0.12^3^ |  |
| **DBP, mean (mmHg)** | **2019** | 81.67$\pm$9.09 | 81.67$\pm$9.01 | 0.99^3^ | 77.46$\pm$7.84 | 75.88$\pm$7.76 | 0.57^3^ | 74.52$\pm$8.03 | 74.65$\pm$8.71 | 0.67^3^ |
|  | **2023** | 76.53$\pm$8.49 | 79.35$\pm$9.71 | 0.08^3^ | 72.92$\pm$8.98 | 75.77$\pm$7.59 | 0.16^1^ | 71.93$\pm$6.92 | 73.72$\pm$8.18 | 0.14^3^ |
|  | **Diff.** | -5.13 | -2.32 | 0.15^1^ | -4.54 | -0.12 | 0.06^1^ | -2.59 | -0.93 | 0.12^3^ |
|  | ***p* value** | 0.005^3^ | 0.06^3^ |  | 0.028^3^ | 0.94^3^ |  | 0.02^3^ | 0.60^3^ |  |
| **FBG, mean (mg/dL)** | **2019** | 100.77$\pm$8.16 | 100.83$\pm$11.75 | 0.39^3^ | 110.81$\pm$16.02 | 110.77$\pm$15.79 | 0.99^1^ | 100.68$\pm$10.22 | 101.82$\pm$13.34 | 0.94^3^ |
|  | **2023** | 96.83$\pm$10.95 | 97.4$\pm$11.28 | 0.57^3^ | 111.85$\pm$22.00 | 111.10$\pm$21.45 | 0.74^3^ | 97.72$\pm$9.85 | 99.72$\pm$22.18 | 0.67^3^ |
|  | **Diff.** | -3.93 | -3.43 | 0.65^3^ | 1.04 | 0.33 | 0.88^2^ | -2.84 | -2.10 | 0.53^3^ |
|  | ***p* value** | 0.02^3^ | 0.001^3^ |  | 0.80^3^ | >0.99^3^ |  | 0.03^3^ | <0.001^3^ |  |
| **HbA1c, mean (%)** | **2019** | 5.54$\pm$0.30 | 5.52$\pm$0.40 | 0.32^3^ | 5.98$\pm$0.49 | 5.99$\pm$0.49 | >0.99^3^ | 5.57$\pm$0.40 | 5.58$\pm$0.46 | >0.99^3^ |
|  | **2023** | 5.60$\pm$0.29 | 5.64$\pm$0.46 | 0.54^3^ | 6.15$\pm$0.64 | 6.17$\pm$0.90 | 0.74^3^ | 5.66$\pm$0.41 | 5.70$\pm$0.60 | 0.74^3^ |
|  | **Diff.** | 0.06 | 0.12 | 0.22^1^ | 0.17 | 0.18 | 0.55^3^ | 0.09 | 0.12 | 0.51^3^ |
|  | ***p* value** | 0.20^3^ | <0.001^3^ |  | 0.15^3^ | 0.50^3^ |  | 0.01^3^ | <0.001^3^ |  |
| **BMI, mean (kg/m^2^)** | **2019** | 26.04$\pm$3.80 | 25.04$\pm$3.22 | 0.34^3^ | 26.99$\pm$2.67 | 25.17$\pm$2.62 | 0.005^1^ | 25.17$\pm$2.06 | 25.17$\pm$2.05 | 0.99^3^ |
|  | **2023** | 25.79$\pm$3.49 | 24.90$\pm$3.01 | 0.39^3^ | 26.95$\pm$3.02 | 24.82$\pm$2.58 | 0.002^1^ | 25.33$\pm$2.25 | 25.36$\pm$2.07 | 0.86^3^ |
|  | **Diff.** | -0.25 | -0.14 | 0.66^3^ | -0.04 | -0.35 | 0.58^2^ | 0.16 | 0.19 | 0.80^3^ |
|  | ***p* value** | 0.472^3^ | 0.45^1^ |  | 0.90^1^ | 0.48^1^ |  | 0.32^1^ | 0.04^3^ |  |
| **Triglycerides,  mean (mg/dL)** | **2019** | 158.57$\pm$76.74 | 152.97$\pm$76.35 | 0.70^3^ | 158.23$\pm$84.33 | 180.17$\pm$86.27 | 0.25^3^ | 172.80$\pm$91.11 | 142.39$\pm$89.49 | 0.02^3^ |
|  | **2023** | 160.47$\pm$126.53 | 149.78$\pm$85.24 | 0.99^3^ | 131.54$\pm$51.17 | 145.77$\pm$75.14 | 0.62^3^ | 132.59$\pm$59.22 | 150.35$\pm$91.56 | 0.58^3^ |
|  | **Diff.** | 1.9 | -3.18 | 0.60^3^ | -26.69 | -34.40 | 0.70^2^ | -40.20 | 7.97 | <0.001^3^ |
|  | ***p* value** | 0.19^3^ | 0.43^3^ |  | 0.06^3^ | 0.03^3^ |  | <0.001^3^ | 0.33^3^ |  |
| **Total Cholesterol, mean (mg/dL)** | **2019** | 189.33$\pm$37.58 | 194.18$\pm$30.29 | 0.16^3^ | 194.23$\pm$43.08 | 201.42$\pm$35.55 | 0.44^1^ | 207.59$\pm$34.40 | 191.02$\pm$33.30 | 0.009^1^ |
|  | **2023** | 180.67$\pm$31.27 | 197.90$\pm$41.99 | 0.06^3^ | 177.73$\pm$48.09 | 194.83$\pm$42.45 | 0.11^1^ | 203.48$\pm$43.29 | 191.59$\pm$34.14 | 0.09^1^ |
|  | **Diff.** | -8.67 | 3.71 | 0.45^3^ | -16.50 | -6.60 | 0.48^1^ | -4.11 | 0.57 | 0.56^3^ |
|  | ***p* value** | 0.98^3^ | 0.46^1^ |  | 0.11^1^ | 0.43^1^ |  | 0.57^1^ | 0.88^1^ |  |
| **HDL Cholesterol,  mean (mg/dL)** | **2019** | 55.50$\pm$15.63 | 52.95$\pm$14.10 | 0.45^3^ | 46.96$\pm$11.24 | 51.62$\pm$12.00 | 0.11^3^ | 53.36$\pm$14.29 | 54.59$\pm$14.06 | 0.47^3^ |
|  | **2023** | 57.87$\pm$13.78 | 56.40$\pm$15.02 | 0.52^3^ | 51.85$\pm$11.61 | 55.98$\pm$11.81 | 0.15^1^ | 59.18$\pm$12.14 | 55.27$\pm$12.24 | 0.09^1^ |
|  | **Diff.** | 2.37 | 3.45 | 0.80^3^ | 4.88 | 4.37 | 0.85^2^ | 5.82 | 0.68 | <0.001^1^ |
|  | ***p* value** | 0.22^1^ | 0.002^3^ |  | 0.02^1^ | 0.03^1^ |  | <0.001^3^ | 0.42^1^ |  |
| **LDL Cholesterol,  mean (mg/dL)** | **2019** | 125.10$\pm$32.54 | 133.35$\pm$28.52 | 0.09^3^ | 137.96$\pm$42.62 | 139.44$\pm$36.56 | 0.87^1^ | 142.32$\pm$30.35 | 130.01$\pm$32.37 | 0.02^3^ |
|  | **2023** | 109.97$\pm$32.91 | 128.37$\pm$35.98 | 0.02^1^ | 116.54$\pm$43.76 | 126.88$\pm$42.23 | 0.32^1^ | 133.18$\pm$41.80 | 122.32$\pm$32.22 | 0.14^2^ |
|  | **Diff.** | -15.13 | -4.98 | 0.50^3^ | -21.42 | -12.56 | 0.52^1^ | -9.14 | -7.69 | 0.88^3^ |
|  | ***p* value** | 0.21^3^ | 0.29^1^ |  | 0.04^1^ | 0.13^1^ |  | 0.36^3^ | 0.03^1^ |  |

^1^ Independent t-test for between-group comparisons under normality and equal variance assumptions; Paired t-test for within-group comparisons under normality. ^2^ Welch t-test for between-group comparison under normality and unequal variances. ^3^ Wilcoxon rank-sum test for between-group comparison and Wilcoxon signed-rank test for within-group comparisons under non-normality.
* Abbreviations: SBP: systolic blood pressure; DBP: diastolic blood pressure**;** FBG: fasting blood glucose; HbA1c: hemoglobin A1c; BMI: body mass index; HDL: high-density lipoprotein; LDL: low-density lipoprotein

**Supplementary Table S3. Short-term changes in lifestyle behaviors between baseline (2019) and follow-up (2023) in the intervention group.**

| **Cohort** | **Lifestyle Behavior** | **Change summary** | | | |
| --- | --- | --- | --- | --- | --- |
| **HTN (N=30)** | **Smoking**;  Current smokers N=7 | 1 participant reduced cigarette consumption per day, while the others showed no change. | | | |
|  | **Alcohol consumption** (drinks/day) | 11 decreased, 6 increased, and the others showed no change. | | | |
|  | **Physical activity** | Post  Pre | ≤150 min/week | <180 min/week | ≥180 min/week |
|  |  | ≤150 min/week | 9 | 3 | 5 |
|  |  | <180 min/week | 4 | 1 | 1 |
|  |  | ≥180 min/week | 1 | 0 | 6 |
| **DM (N=25)** | **Smoking**;  Current smokers N=10 | 3 participants reduced cigarette consumption per day, while the others showed no change. | | | |
|  | **Alcohol consumption** (drinks/day) | 11 decreased, 6 increased, and the others showed no change. | | | |
|  | **Physical activity** | Post  Pre | ≤150 min/week | <180 min/week | ≥180 min/week |
|  |  | ≤150 min/week | 7 | 3 | 2 |
|  |  | <180 min/week | 2 | 2 | 1 |
|  |  | ≥180 min/week | 0 | 2 | 6 |
| **Obesity (N=41)** | **Smoking**;  Current smokers N=5 | 3 participants reduced cigarette consumption per day, while the others showed no change. | | | |
|  | Alcohol consumption (drinks/day) | 19 decreased, 10 increased, and the others showed no change. | | | |
|  | Physical activity | Post  Pre | ≤150 min/week | <180 min/week | ≥180 min/week |
|  |  | ≤150 min/week | 10 | 1 | 5 |
|  |  | <180 min/week | 2 | 5 | 3 |
|  |  | ≥180 min/week | 2 | 6 | 7 |

* Data were collected from participants who completed both pre- and post-intervention questionnaires.
